# The Book World of Medicine and Science

**Published:** 1900-02-17

**Authors:** 


### THE BOOK WORLD OF MEDICINE AND SCIENCE.

Twentieth Century Practice: An International Encyclopædia of Modern Medical Science. Edited by Thomas L. Stedman, M.D. Volume XVIII. (London: Sampson Low, Marston, and Co. 1899.)

The eighteenth volume of this great work is entirely occupied by two subjects, syphilis and leprosy, its treatment of both of which is complete and authoritative. The main bulk of the volume is devoted to the article by Professor Eduard Lang, of Vienna, on "Acquired Syphilis," and that of Professor Prince A. Morrow, of New York, on "Leprosy." Sandwiched between these is one of thirty pages or so by Mr. Jonathan Hutchinson on "Inherited Syphilis," which, although short, is full. In the 370 pages which he devotes to the subject, Professor Lang gives a remarkably complete and practical description of acquired syphilis in all its various stages and degrees. The descriptive part is encyclopædic rather than clinical, and although every possible mode in which the disease is likely to occur is described, there seems a certain lack of proportion in the amount of importance attributed to its various manifestations. The strong part of the article is that devoted to treatment, which is very complete and full of useful suggestions. On entering upon this subject Professor Lang says that he wishes to emphasise the fact that the virus which has entered the organism may die out spontaneously, and that syphilis may therefore be cured occasionally without the intervention of drugs. The possibility of this spontaneous recovery taking place has, he says, led some to leave syphilis to its spontaneous development, and thus to reduce treatment to a simple matter of regimen—the "simple treatment." But it is to be noted that many even of the advocates of this "simple treatment" have been forced to return to mercury, because they have found that syphilis by no means always runs its course benignly. Lang says that although he is convinced that, in the great majority of cases, lues runs a comparatively mild course, "we are not justified in a policy of inaction, for the simple reason that in any individual case we have no data upon which to base a positive prognosis or the further course of the disease." On the other hand, as against undergoing a full course of treatment, "it must be honestly admitted that while we can probably in the great majority of cases not only cause the disappearance of the symptoms but also prevent the transmission of the disease by heredity, yet up to the present time we do not possess any mode of treatment by which we can positively cure this dangerous disease radically and thus absolutely protect the patient against relapses." Nevertheless, for our comfort it is stated that by the rational use of the means at our disposal we are able in numberless cases to effect a perfect cure of syphilis, as shown by the patient and his offspring remaining free from all symptoms, and especially by the former occasionally becoming reinfected. In regard to the use of mercury in the treatment of syphilis Lang places the different methods of administering in the following order of preference. First, subcutaneous injections of grey oil, calomel, thymolate, and saliculate of mercury; and in the second rank injections of soluble mercurial preparations; next in order come inunctions, smearing, the use of mercurial soaps, plaster treatment, and electric corrosive sublimate baths; and, finally, internal treatment. He points out, however, that one may not infrequently be limited in one's choice by the external circumstances of the patient. A very useful section of this chapter is devoted to a consideration of the methods by which the dangers of mercurialism may be prevented. In regard to the use of iodides, Lang says that, although, as a rule, the early forms of syphilis react only very little to iodine, we nevertheless occasionally find that early relapses are markedly improved by it, and also that it proves very effective in relieving the neuralgic pains in the bones and joints which precede or accompany the early eruption. He also gives iodides in scrofulous and debilitated subjects, and in those cases in which from the first the disease has taken on a malignant course, although when the health has improved these patients may still require to undergo mercurial treatment.

The article by Mr. Hutchinson on inherited syphilis is very full of information, and is one to which practitioners may well refer in regard to various disputed points. Among other matters Mr. Hutchinson draws attention to the danger of causing injury to the second set of teeth by administering mercury in infancy, and cites this as a reason for not administering it unless absolutely necessary. He says: "If an infant known to have inherited syphilis displays no symptoms, and is in good general health, it is better to abstain from treatment. By doing so it may be thought that we increase the risk of developments at a more advanced age, such as keratitis, &c. This, however, is a matter by no means certain, whereas the damage to the incisor and molar teeth is inevitable." It may be mentioned in passing that Mr. Hutchinson "utterly dissents" from the statement that syphilis is likely, by its inheritance, to become a cause of degeneracy of race; and thinks that the extent to which its hereditary transmission influences the well-being of the community is exceedingly small.

The article on leprosy by Professor Morrow gives a very careful review of our knowledge regarding this curious disease, which troubles us so little, and yet is so serious a burden in many other countries. Among the general conclusions at which he arrives are—that leprosy is in the vast majority of cases an incurable disease; that all treatment which has for its object the destruction of the bacilli is impossible of application; that among the empirical remedies which have been employed, those which from clinical evidence seem of most value are chaulmoogra oil, gurjun oil, and certain agents of the strychnos family; that serum treatment has not fulfilled the expectations of its value, and that tuberculin has been disappointing; that in some cases the resistance of the tissues is such that signs of the disease disappear and never recur, and that in strengthening this power of resistance by change of climate, by improved habits of living, and by maintaining the health at the highest standard, lies the best chance of giving relief in cases seen in the early stages. Even by these means, however, cure is very doubtful, for among the 168 Norwegian lepers who have emigrated to the United States there is no record of a single definite cure. In regard to prophylaxis much is said upon both sides regarding the question of segregation, with this outcome that, although the author believes that segregation is the most effective measure that can be employed to limit the spread of leprosy, and thus in some countries is a necessary measure, he does not believe that it can ever result in the entire suppression of the disease. Much historical information is given in this article, and a good deal that will be found interesting in regard to the geographical distribution of leprosy.

